# Euglena International Network (EIN): Driving euglenoid biotechnology for the benefit of a challenged world

**DOI:** 10.1242/bio.059561

**Published:** 2022-11-22

**Authors:** ThankGod Echezona Ebenezer, Ross S. Low, Ellis Charles O'Neill, Ishuo Huang, Antonio DeSimone, Scott C. Farrow, Robert A. Field, Michael L. Ginger, Sergio Adrián Guerrero, Michael Hammond, Vladimír Hampl, Geoff Horst, Takahiro Ishikawa, Anna Karnkowska, Eric W. Linton, Peter Myler, Masami Nakazawa, Pierre Cardol, Rosina Sánchez-Thomas, Barry J. Saville, Mahfuzur R. Shah, Alastair G. B. Simpson, Aakash Sur, Kengo Suzuki, Kevin M. Tyler, Paul V. Zimba, Neil Hall, Mark C. Field

**Affiliations:** ^1^ European Molecular Biology Laboratory, European Bioinformatics Institute, Wellcome Genome Campus, Hinxton, Cambridge CB10 1SD, UK; ^2^ Organisms and Ecosystems, Earlham Institute, Norwich Research Park, Norwich NR4 7UZ, UK; ^3^ School of Chemistry, University of Nottingham, Nottingham NG7 2RD, UK; ^4^ Office of Regulatory Science, United States Food and Drug Administration, Center for Food Safety and Applied Nutrition, College Park, MD 20740, USA; ^5^ The BioRobotics Institute, Scuola Superiore Sant'Anna, Pisa 56127, Italy; ^6^ Discovery Biology, Noblegen Inc., Peterborough, Ontario K9L 1Z8, Canada; ^7^ Environmental and Life Sciences Graduate Program, Trent University, Peterborough, Ontario K9L 0G2, Canada; ^8^ Department of Chemistry and Manchester Institute of Biotechnology, University of Manchester, Manchester M1 7DN, UK; ^9^ School of Applied Sciences, University of Huddersfield, Huddersfield HD1 3DH, UK; ^10^ Laboratorio de Enzimología Molecular, Instituto de Agrobiotecnología del Litoral. CCT CONICET Santa Fe, Santa Fe 3000, Argentina; ^11^ Institute of Parasitology, Biology Centre, Czech Academy of Sciences, České Budějovice 370 05, Czech Republic; ^12^ Charles University, Faculty of Science, Department of Parasitology, BIOCEV, Vestec 25250, Czech Republic; ^13^ Kemin Industries, Research and Development, Plymouth, MI 48170, USA; ^14^ Institute of Agricultural and Life Sciences, Academic Assembly, Shimane University, Matsue 690-8504, Japan; ^15^ Institute of Evolutionary Biology, Faculty of Biology, University of Warsaw, Warsaw 02-089, Poland; ^16^ Department of Biology, Central Michigan University, Mt. Pleasant, MI 48859, USA; ^17^ Center for Global Infectious Disease Research, Seattle Children's Research Institute and Department of Biomedical Informatics & Medical Education, University of Washington, WA 98109, USA; ^18^ Department of Applied Biochemistry, Faculty of Agriculture, Osaka Metropolitan University, Sakai, Osaka, 599-8531, Japan; ^19^ Department of Life Sciences, Institut de Botanique, Université de Liège, Liège 4000, Belgium; ^20^ Instituto Nacional de Cardiología, Ignacio Chávez, Mexico 14080, Mexico; ^21^ Forensic Science, Environmental and Life Sciences Graduate Program, Trent University, Peterborough K9L 0G2, Canada; ^22^ Department of Biology and Institute for Comparative Genomics, Dalhousie University, Halifax, Nova Scotia B3H 4R2, Canada; ^23^ R&D Company, Euglena Co., Ltd., 2F Yokohama Bio Industry Center (YBIC), 1-6 Suehiro, Tsurumi, Yokohama, Kanagawa, 230-0045, Japan; ^24^ Biomedical Research Centre, Norwich Medical School, University of East Anglia, Norwich Research Park, Norwich NR4 7TJ, UK; ^25^ Center of Excellence for Bionanoscience Research, King Abdul Aziz University, Jeddah, Saudi Arabia; ^26^ PVZimba, LLC, 12241 Percival St, Chester, VA 23831, USA; ^27^ Rice Rivers Center, VA Commonwealth University, Richmond, VA 23284, USA; ^28^ School of Biological Sciences, University of East Anglia, Norwich, NR4 7TJ, Norfolk, UK; ^29^ School of Life Sciences, University of Dundee, Dundee DD1 5EH, UK

**Keywords:** Euglena, Networks, Biotechnology, Biofuels, Food supplements, Bioremediation

## Abstract

Euglenoids (Euglenida) are unicellular flagellates possessing exceptionally wide geographical and ecological distribution. Euglenoids combine a biotechnological potential with a unique position in the eukaryotic tree of life. In large part these microbes owe this success to diverse genetics including secondary endosymbiosis and likely additional sources of genes. Multiple euglenoid species have translational applications and show great promise in production of biofuels, nutraceuticals, bioremediation, cancer treatments and more exotically as robotics design simulators. An absence of reference genomes currently limits these applications, including development of efficient tools for identification of critical factors in regulation, growth or optimization of metabolic pathways. The Euglena International Network (EIN) seeks to provide a forum to overcome these challenges. EIN has agreed specific goals, mobilized scientists, established a clear roadmap (Grand Challenges), connected academic and industry stakeholders and is currently formulating policy and partnership principles to propel these efforts in a coordinated and efficient manner.

## Teaser

**Summary:** Euglenoids show great promise to benefit our world; as biofuels, robotics design simulators, anti-cancer and food nutritional agents, but the absence of reference genomes currently limit realizing these benefits.

## Background

The contributions of fungi and bacteria towards many global needs, including the generation of foodstuffs, pharmaceuticals and energy are well known, ancient and remain an ongoing challenge to improve. Non-fungal unicellular eukaryotes (protists), represent a comparatively untapped potential resource, and with a significant range of lifestyles, considerable opportunity. This lack of exploration arises from a combination of factors: absence of genetic tractability, well defined culture conditions, incomplete understanding of genomic potential and, in many cases, undersampling of the taxa. A specific example, and the subject of the current article, are potential benefits from exploiting euglenoids. Such potential is frequently well known but under-exploited or remains as a niche product, such as nutrient supplementation in Japan. The Euglena International Network (EIN) seeks to provide a forum to overcome such challenges for euglenoids in particular, of which there are multiple examples of potential.

Multiple euglenoid species have translational applications and show great promise in biofuls, nutraceuticals, bioremediation, cancer treatments and more exotically as robotics design simulators (Table 1). But the absence of reference genomes currently limits these applications, including development of efficient tools for genome-wide screens for identification of critical factors in regulation, growth or optimization of metabolic pathways. Furthermore, this has also inhibited the development of genetic tools, including CRISPR-mediated gene editing, for the precise engineering of euglenoids for exploration. There are nearly 1000 known euglenoid species (Triemer and Zakryś, 2015), but fewer than 20 have been explored at any level for translational potential through genomics, albeit that several recent advances are beginning to address these obstacles. EIN (https://euglenanetwork.org) aims to address these and other challenges.

**Table 1.**
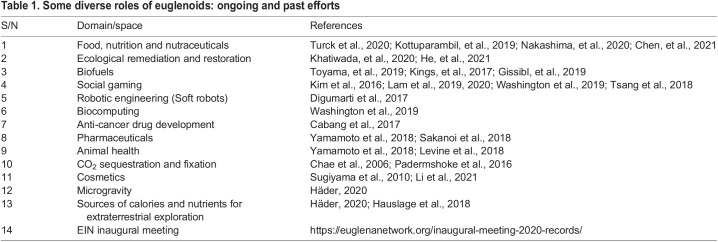
Some diverse roles of euglenoids: ongoing and past efforts

Advancement of euglenoid science through a coalition of academic institutions, national research institutes and the biotechnology industry requires genome sequence data, as does translating and exploiting these organisms in an efficient manner. EIN has defined goals, mobilized scientists, established a clear roadmap (Grand Challenges), connected academic and industry stakeholders, and is currently formulating policy and partnership principles to propel these efforts in a coordinated and efficient manner. This program is underpinned by EIN Executive and Science committees, with early-career researchers (ECRs) driving EIN's agenda, as recently demonstrated at the EIN's international conference (Kaszecki, et al., 2022). However, for EIN's activities to be maintained and durable, long-term support is vital. We call on national and supranational funding agencies, protist and algae scientific communities, and the biotechnology and pharmaceutical industries to embrace and resource EIN and to support translational exploitation of a valuable resource in especially challenging times.

## About the euglenoids

**Fig. 1.**
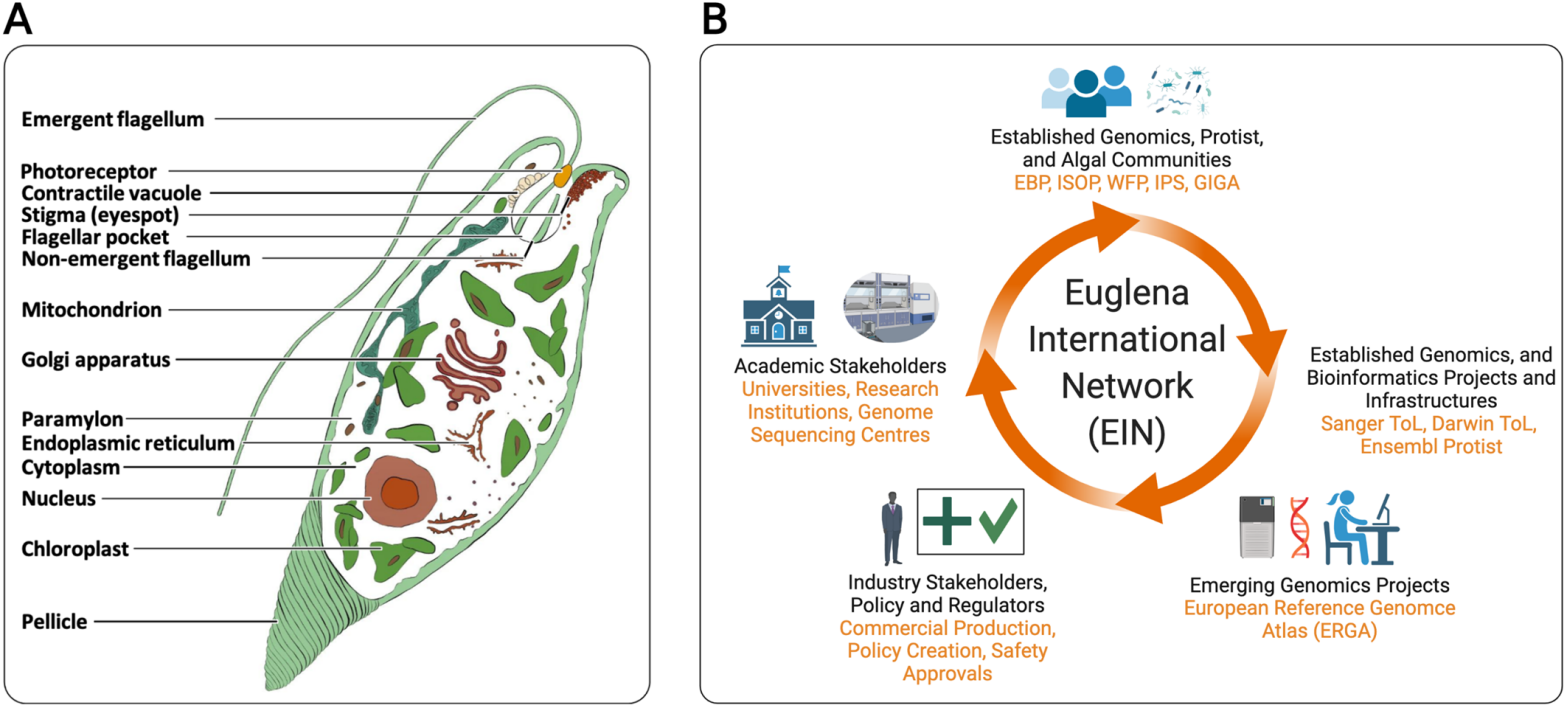
**(A) *Euglena gracilis* is a unicellular organism with the canonical complement of internal cellular organelles for a photosynthetic protist, with one to two emergent flagella.** An obvious morphological feature unique to euglenoids is the pellicle, a series of interlocking protein strips below the plasma membrane. Examples of characteristic metabolic features include paramylon, a β-1,3 glucan and the production of combustible wax esters. (B) EIN aims to meet challenges and ambitions through multi -institutional, -country and -sector partnerships. Earth BioGenome Project (EBP), International Society of Protistologists (ISOP), World Federation of Parasitology (WFP), International Phycological Society (IPS), Phycological Society of the Americas, Global Invertebrates Genomics Alliance, Sanger Tree of Life (Sanger ToL), Darwin Tree of Life (DToL) and European Reference Genome Atlas (ERGA) are all identified, prospective EIN partners.

**Fig. 2.**
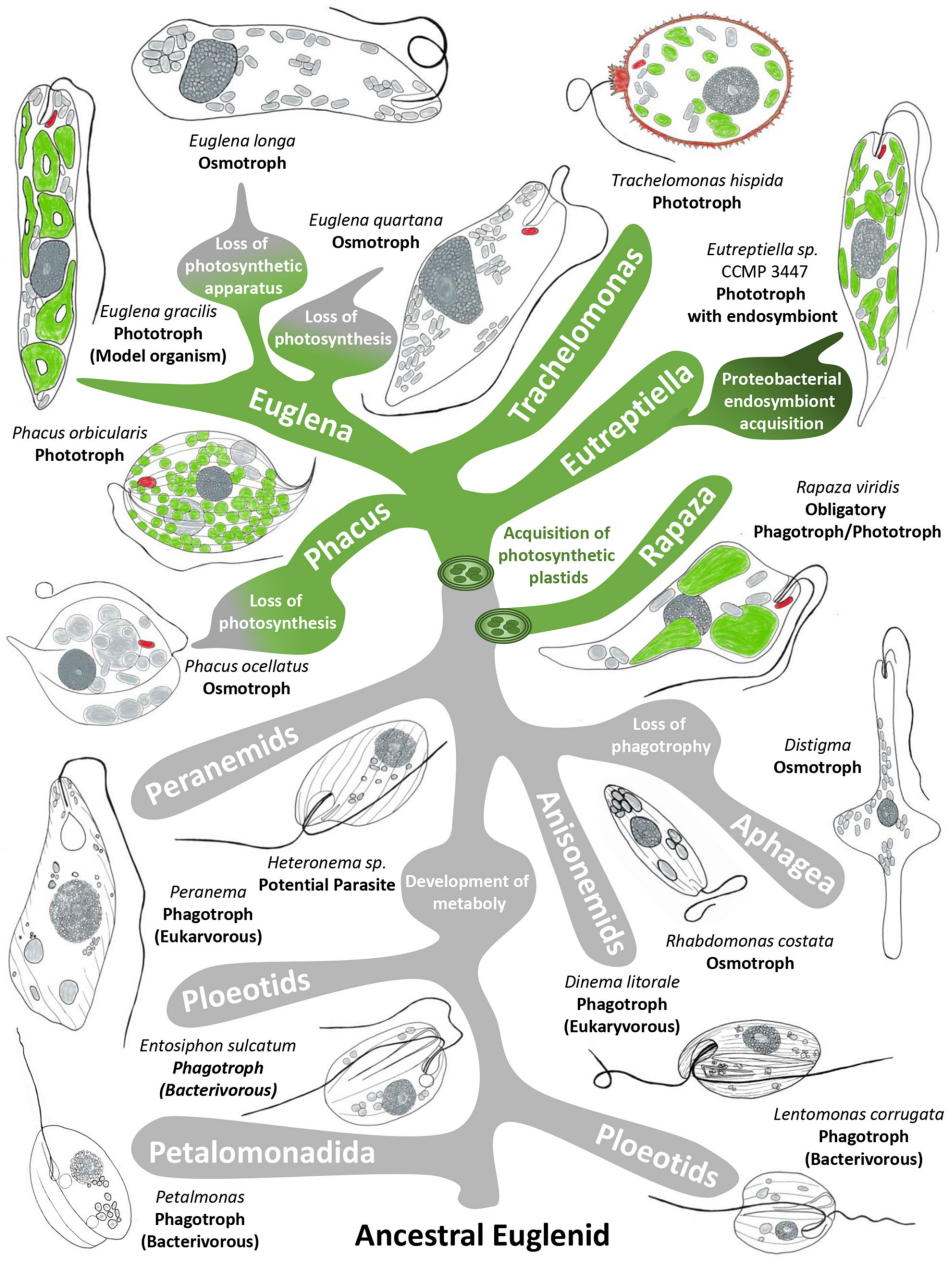
**Simplified euglenoid phylogenetic tree.** Selected branching from the last euglenoid common ancestor (LEUCA) showing important evolutionary developments and groups exhibiting various life strategies. Acquisition of the photosynthetic lifestyle is represented through green branches, with dark green representing the further acquisition of bacterial endosymbionts. Phototrophic plastids within euglenoids are depicted in green, with a light-shielding stigma as well as lorica for *Trachelomonas hispida* depicted in red. Relationships among euglenoids according to Lax et al. (2021).

Euglenoids (Euglenida) are unicellular flagellates with an exceptionally wide geographical and ecological distribution, including aquatic and terrestrial ecological niches (Leander, et al., 2017; Kostygov, et al., 2021). Euglenoids, in large part, owe this success to a diverse genetic repertoire including secondary endosymbiosis with a green alga (Dorrell et al., 2017). *Euglena gracilis* (Figs 1A and 2) remains the most characterized representative, due to the combination of interests; its status as a model organism, its evolutionary and taxonomic position, societal benefits and significant biotechnological potential (Zoltner and Field, 2022, in press). An incomplete draft genome of up to 3Gb, with nearly 40, 000 protein coding genes and additional complexity due to alternate splicing, potentially underpins the wealth of natural products *E. gracilis* makes (Ebenezer, et al., 2019). These combined basic science and translational aspects of euglenoids can be maximized and more fully explored, but are limited by the primitive nature of our current genetic tools (Harada, et al., 2020), in part due to the overall genome size, heterozygosity, hyper-modified nucleotides and a high frequency of repetitive sequences.

## Opportunities and challenges

It is rare to encounter an organism so versatile that it offers possible roles in food production, biofuel generation, bioremediation, social gaming, robotic engineering, biocomputing, drug development, beauty products, animal health, CO_2_ sequestration and a source of calories and nutrients for extraterrestrial exploration. Euglenoids naturally possess features supporting these biotechnological applications within their unmodified genomes and cells (Table 1). For example, euglenoids produce dozens of molecules including vitamins (e.g. A through to E), polysaccharides (e.g. paramylon), lipids (saturated, mono- and polyunsaturated fatty acids) and bioactive natural products like euglenophycin (Zimba et al., 2017; Cabang et al., 2017; Gissibl, et al., 2019) and euglenatides (Aldholmi et al., 2022). This differentiates euglenoids from many biotechnology chassis, such as bacteria and fungi, where extensive modifications are frequently required to produce products of sufficient value. Similarly, in the face of climate change, euglenoids have demonstrated extraordinary efficiency in absorbing CO_2_ emissions, acting in both carbon capture and product synthesis capacities (Table 1).

There have been several advances in the area of single-cell analyses that are driving our understanding of euglenoids (i.e. *Euglena gracilis*) and their advancement into the bioeconomy. This includes frequency-division multiplexing (FDM) confocal fluorescence microscopy (Mikami et al., 2018), imaging flow cytometry (Mikami et al., 2020), label-free video-rate metabolite imaging (Wakisaka et al., 2016), and single-cell mass spectrometry (Cahill et al., 2019). But implementing these techniques can be challenging. For example, it is traditionally challenging to perform 3D fluorescence imaging of motile organisms like *Euglena gracilis* because of their dynamic motion. However, FDM confocal fluorescence microscopy has an exceptionally high capture rate, and when employed on *Euglena,* enables high-resolution imaging of their 3D dynamic motion (Mikami et al., 2018). It will be interesting to see how this technique could be paired with genetic engineering, image and machine learning for identifying valuable euglenoids, feed algorithms or fermentation conditions to ensure desired cellular composition.

## Why a network?

Recognizing the challenges to Euglenoid research and the huge potential, in 2020 an international group of ECRs founded the Euglena International Network (EIN) (https://euglenanetwork.org), which currently has 23 committee members and 215 non-committee members. These ECRs form the EIN executive committee, including representatives from industry, universities and research institutes, and receive support and advice from an advisory committee of senior experts and a science committee who possess a range of specialties. EIN aims to coordinate the power of euglenoid science to exploit the biotechnology potential on offer (Table 1). Since the inaugural meeting in November 2020, EIN has organized an annual online conference (Kaszecki et al., 2022), with an upcoming meeting scheduled for November 2022, stressing inclusion of researchers from diverse backgrounds and all career stages.

Since its inception EIN has focused on formulating a partnership framework, scoping the Euglenoid Genomes Project (EGP), which aims to sequence the genomes of nearly 1000 euglenoids, and establishing policy directives for governance. For instance, EIN has connected with prospective industry partners, including Euglena Co. Ltd., Kemin Industries, and Noblegen Inc., as well as not-for-profit research centers and initiatives, such as the Earlham Institute (EI), Manchester Institute of Biotechnology (MIB), Darwin Tree of Life (DToL), and the European Bioinformatics Institute (e.g. Ensembl Protist and the Universal Protein Resource, UniProt) (Fig. 1B). EIN plans to bring together 500 scientists, industry professionals, stakeholders, and partners, to supercharge euglenoid research, initially focusing on sequencing euglenoid genomes that span the breadth of euglenoid diversity, and translating the data generated into societal benefits.

## Why the EIN goals matter

EIN plans to generate high quality reference genomes of the nearly 1000 euglenoids species in ten years, which will guide four critical future directions for euglenoid science and technology development:

*1. Exploration, translation and commercialisation of euglenoid products*: the key bottleneck in product development (e.g. food, nutrition, nutraceuticals) is the availability of high-value bioactive ingredients, molecules, and nutritional components capable of long-term potency. Euglenoids can contribute towards the solution for industrial bioactive ingredient-based problems in the global biotechnology industry, with a market size of USD 177.7 billion in 2019 and expected to reach USD 298.54 billion by 2027. For instance, Euglena Co. Ltd. has commercialized human superfoods from *E. gracilis*, supplied biodiesel refined from Euglena wax esters and aims to produce the first certified algae jet fuel blend (Table 1). Similarly, Kemin Industries has economically produced *E. gracilis* biomass, rich in the immune modulating glycopolymer paramylon (beta-1,3-glucan) with clinical trials suggesting human immune, gastrointestinal, and even mental health and sleep benefits (Table 1). Finally, Noblegen Inc. is leveraging *Euglena* for water filtration and consumer facing foods (Eunite Foods), including protein-rich *Euglena* flour and *Euglena* meat analogues (such as simulated chicken nuggets, pulled pork, smokey tuna) (Table 1).
  In *Chlamydomonas reinhardtii* starch (polymeric α-1,4-glucose) is the major carbon store (Burlacot, et al., 2019), but in *Euglena gracilis* the storage form is paramylon, with β-1,3-glycosidic linkage (Gissibl et al., 2019; Ruiz-Herrera and Ortiz-Castellanos, 2019; Haslam et al., 2022; Singdevsachan et al., 2016). Starch is amorphous and water soluble but paramylon is water insoluble and accumulates as cytoplasmic granules (Kiss et al., 1988; Monfils et al., 2019); these forms are suitable for facile extraction to purity exceeding 90%. Further, β-glucans are well-known immune modulatory molecules, present in both euglenoids and yeasts (Nakashima et al., 2018; Evans, et al., 2019). In yeast glucans are challenging, expensive and inefficient to isolate as they are bound to the cell wall (Avramia and Amariei, 2021), but in euglenoids glucans are *freely* present in the cytoplasm, offering an excellent route to highly pure products (Levine et al., 2017; O'Neill et al., 2015a,b; Gissibl et al., 2019; Watanabe et al., 2013; Wang et al., 2018).
  To date, translation and exploitation of euglenoids has focused on one species, *E. gracilis.* To maximize the full suite of potentials and advance market acceptance for more of the nearly 1000 euglenoid species, translation and commercialisation of euglenoid research must be accelerated, including scaling and supporting crowdfunding initiatives to carry out basic toxicological studies on generic strains of euglenoids crucial to easing regulatory hurdles. For example, this can be seen in the rigorous safety evaluation on BetaVia Complete; an expensive, multi-year process undertaken by Kemin to obtain a European Food Safety Agency (EFSA) food permit for whole-cell *E. gracilis* (Table 1)*.*
*2. Maximize euglenoid applications in ecological and environmental management*: a key bottleneck in bioremediation of contaminated and polluted environments using living systems is availability of efficient biological systems able to sequester pollutants without accumulating in the food chain (Moreno-Sánchez et al., 2017). For example, the presence of euglenoids, and the ichthyotoxin euglenophycin produced by *E. sanguinea* (Zimba et al., 2017), can act as sentinels for environmental changes. However, *E. sanguinea* lacks a reference genome to inform on controlling conditions for environmental management and biomonitoring. Therefore, EIN proposes to include several transcriptome environmental reference data sets (for example, high and low temperature, light and dark conditions) as part of the EGP to fully capture expressed genes under different environmental and laboratory controlled conditions.
*3. Understand the basic biology of euglenoids*: ploidy, mechanisms of DNA recombination and the sexual habits of *E. gracilis* remain unknown but are critical as a prelude to sophisticated, robust forward and reverse genetics. For example, the current draft genome of *E. gracilis* suggests a polyploid genome configuration (Ebenezer et al., 2019) albeit with some studies suggesting a haploid state (Ebenezer et al., 2017). Molecular reconstructions of the evolutionary histories of euglenoid metabolism, gene regulation and how these protists perceive environmental cues will provide the fundamental basis for further commercializing euglenoid biology or developing euglenoid biosensors. EIN will develop robust forward and reverse genetics such as gene silencing (Nakazawa et al., 2015; Muchut et al., 2021) and gene knockout (Nomura et al., 2020) for several euglenoid species.
*4. Understand the evolution of euglenoids*: euglenoids are related to the kinetoplastids (*trypanosomes*, *Leishmania*) and the diplonemids, on the evolutionary tree of life (Keeling, 2004; Kostygov, et al., 2021). Kinetoplastids are parasites of vertebrates, insects and plants, and cause immeasurable suffering and commercial loss across much of the globe. The study of euglenoids will help to expedite basic research against parasitic diseases such as trypanosomiasis (sleeping sickness) and leishmaniasis. For instance, investigations into *Trypanosoma brucei* mitochondria have unearthed a variety of diverged core structures including respiratory subunits (OXCT2 and NDUFS3) (Pagliarini, et al., 2008), protein import machinery (ATOM40, pATOM36 and TIM22) (Schneider, 2018) and the mitochondrial contact site and cristae organization system (subunits Mic20 and Mic34) (Kaurov et al., 2018), all of which represent exciting medicinal candidates due to their importance for organism viability as well potentially being lineage-specific. EIN will identify several basal euglenoid species and establish their cultivation for detailed cell and genetic study, with particular focus on heterotrophic representatives with varied nutritional modalities (Fig. 2).

The last euglenoid common ancestor (LEUCA) was likely phagotrophic, with many species of the earliest euglenoid branches (Petalomonadida, ploeotids) being bacterivores. The euglenoids that branched after the putative origin of metaboly movement (peranemids, anisonemids) are generally larger and primarily prey on other eukaryotes. At least one branch of euglenoids lost the ability to perform phagocytosis (Aphagea) and are osmotrophic feeders, referred to as ‘primary osmotrophs’. One specimen of the peranemid branch (*Heteronema* sp.) was identified alive within *Gastrotricha* intestines and may represent a parasitic or commensal lifestyle. Engulfment of algal prey presumably led to acquisition of photosynthetic plastids facilitating phototrophic lifestyles (for example, *Phacus*, *Euglena*, *Trachelomonas*, *Eutreptiella*, *Rapaza*). Some of these euglenoids, ‘secondary osmotrophs’, have lost the ability to employ photosynthesis (*E. longa, E. quartana, Phacus ocellatus*), either through the reduction (*E. longa*) or entire loss of the photosynthetic apparatus. *Eutreptiella* representative CCMP 3447 constitutes one of the few euglenoids known to have additionally acquired bacterial endosymbionts, though the relationship these endosymbionts have with their euglenoids host requires further investigation (Fig. 2).

## A call to action

The production of high-quality reference genomes of euglenoids requires a combination of high-quality genome sequencing platforms with >99% read accuracy such as the current PacBio Hi-Fi technology (Wenger et al., 2019) and Oxford Nanopore. To provide reference genomes for nearly 1000 euglenoid species over the next decade a combination of long-read and short-read will be required, as has been demonstrated by the Vertebrate Genomes Project (VGP) (Rhie et al., 2021). We call on national and regional funding agencies and genomic projects, protist and algal scientific communities (for example, the International Society of Protistologists, ISOP; the International Phycological Society, IPS; and the World Federation of Parasitologists, WFP), biotechnological and pharmaceutical companies, to embrace euglenoids as unique and valuable organisms for their basic sciences and translational processes and to provide formative phase resources to support the objectives of EIN.

## Next steps

EIN will make five bold steps to address the identified Grand Challenges in euglenoid science:

*1. Formation of the Euglenoid Genomes Project (EGP)*: To build the foundation required to deliver on EIN's promises, EIN will progress to initiate the EGP with the goal of exploration and exploitation of the appropriate technologies to produce 1000 reference genomes of euglenoids. These technologies must solve the problems experienced for *E. gracilis* (Ebenezer, et al., 2019), most critically to allow assembly of chromosomal-sized contigs.
*2. Unlock euglenoids potential through an Open EIN*: To maximize scientific contributions and benefits, EIN will adhere to the FAIR principle (findable, accessible, interoperable and reusable) by publishing and depositing all genomic data in open access journals and public databases, such as the international nucleotide sequence database collaboration (INSDC) and Ensembl Protist, respectively. For instance, as part of EGP, EIN plans to develop the Euglenoids Genome Browser, which could be hosted in the Eukaryotic Pathogen Database (EuPathDB) to allow open curation of the genomic data generated, including identifying metabolites, and contributions in generating secondary structures of euglenoid proteins through AlphaFold (Jumper et al., 2021).
*3. Bioprospecting of high-value products, strains and species*: *E. gracilis* produces a plethora of known valuable products (e.g. glycopolymers and small molecules), but we lack a detailed library of potential products (i.e. metabolites, proteins, genes) within *E. gracilis* or euglenoids in general. These products, strains and species could be tremendously important for solving global challenges like disease prophylaxis, treatments, and combating poor nutrition. EIN will exploit modern analytical and omics tools to identify and characterize known euglenoid strains and species, including their natural product profiles and genetic or protein complement.
*4. Developing tools and techniques for genetic interrogation and modification*: we believe that providing open access to EIN's genomic data will accelerate exploration of the plethora of uncharacterized euglenoid genes, a potential treasure trove for the emerging fields of biocatalysis and synthetic biology. For instance, reverse genetics techniques would allow access to the functions of uncharacterized euglenoid genes, and thus, their potential for commercial application and production of high-value compounds and therapeutics.
*5. Build a framework for commercial and academic collaborations*: as biotech companies emerge and use euglenoids in their products, there will be increasing opportunities for academics to synergize with industry. EIN will continue to serve as a platform for realizing potential academic and industry partnerships, whether it be to advance fundamental research or explore commercial products that would benefit many interested parties. For example, researchers that identify and optimize expression of a target molecule (such as tocopherol) could partner with a large-scale industry producer to market and sell the product.

## Conclusion

The tremendous biological potential contained within euglenoids is being overlooked, and at this critical time in global history this needs to be addressed considering euglenoids positions in the eukaryotic tree of life. EIN seeks to meet this challenge, but must be beneficial and open to communities in both developing and developed economies, and for workers at all career stages. Such benefits could be realized in the generation of intellectual property (IP), creation of jobs in new frontiers, training and career development, production and administration of highly nutritious, but cost effective, euglenoid nutrients, ecological tools, fuels and simply the basic fascination with this remarkable group of organisms.
